# Machine learning and atomistic origin of high dielectric permittivity in oxides

**DOI:** 10.1038/s41598-023-49603-2

**Published:** 2023-12-14

**Authors:** Yuho Shimano, Alex Kutana, Ryoji Asahi

**Affiliations:** Nagoya University, Furo-cho, Chikusa-ku, Nagoya, Japan

**Keywords:** Materials for devices, Materials for energy and catalysis, Atomistic models

## Abstract

Discovering new stable materials with large dielectric permittivity is important for future energy storage and electronics applications. Theoretical and computational approaches help design new materials by elucidating microscopic mechanisms and establishing structure–property relations. Ab initio methods can be used to reliably predict the dielectric response, but for fast materials screening, machine learning (ML) approaches, which can directly infer properties from the structural information, are needed. Here, random forest and graph convolutional neural network models are trained and tested to predict the dielectric constant from the structural information. We create a database of the dielectric properties of oxides and design, train, and test the two ML models. Both approaches show similar performance and can successfully predict response based on the structure. The analysis of the feature importance allows identification of local geometric features leading to the high dielectric permittivity of the crystal. Dimensionality reduction and clustering further confirms the relevance of descriptors and compositional features for obtaining high dielectric permittivity.

## Introduction

High dielectric permittivity materials are the key component in capacitive electronic and high electric power density applications and devices^1^. Besides the required high relative dielectric permittivity, the desired properties for such materials include temperature and electric field stability, low dielectric losses, and high breakdown voltage. Most of the presently known materials with highest permittivities do not meet all of these conditions, limiting applications. For example, the high permittivity of ferroelectrics near the phase transition shows large variations with respect to temperature and external electric field. In another kind of materials, CaCu_3_Ti_4_O_12_ being a typical example^2^, the apparent high permittivity arises due to extrinsic effects, e.g. barrier layer capacitance at the grain boundaries^3–5^, making these materials impractical due to the high dielectric loss.

Insulating paraelectrics are free from these shortcomings, and are therefore considered the best candidates for dielectric applications. As the permittivity of the known paraelectrics is only moderate (10–10^2^), the present challenge is to find stable and loss-free paraelectrics with large permittivity > 10^2^. One of the most successful and systematic approaches towards this goal is to increase the intrinsic permittivity of the host paraelectric by permittivity “boosting”^6–8^ through impurity doping. In In-Nb co-doped rutile TiO_2_, permittivity boosting to > 10^4^ was originally reported^9^. Subsequent studies revealed that the major part of this apparent permittivity increase stems from the grain boundary^10–12^ and contact^13^ barrier layer capacitances, just as in CaCu_3_Ti_4_O_12_. More detailed studies^6,7,13^ show however, that at low temperatures where all thermally excited carriers are frozen out and insulating state restored, the rutile permittivity is indeed boosted by co-doping, although the effect is smaller than originally reported. Our previous theoretical analysis^8^ confirmed intrinsic permittivity boosting in co-doped rutile and other substituted paraelectric titanates, and also showed that the effect can be accounted for by the lattice mechanism. It was found that the boosting is due to the softening of the active phonon mode by the local strain^14^ from impurities, and a simple descriptor in the form of the maximum Ti–O bond length was proposed^8^. The descriptor was found heuristically; finding such descriptors is generally challenging and largely depends on luck and intuition. The descriptor is of limited utility as it only applied to titanates; correlation between more general structural features and permittivity should be pursued to apply to the other metal oxides.

In this regard, machine learning (ML) approaches offer a more systematic way of finding relevant descriptors and features in materials, which can also be utilized for property predictions. Here, we use two ML approaches, random forest (RF)^15,16^ and graph convolutional neural network (GCNN)^17,18^, to predict dielectric constants, dynamic stability, and identify features that are relevant to high permittivity. While differing in approaches, both methods can be used for classification and regression tasks. RF is an ensemble method that operates by polling decision trees, while GCNNs can learn complex dependencies on the graph through neighborhood aggregation schemes. RF has been previously employed^19^ for predicting the dielectric constants of oxides found in the Materials Project^20^ database. Being one of the most robust and best performing universal techniques for regression and classification, RF can serve as a benchmark for other ML techniques, such as GCNNs, for dielectric constant prediction. Here we extend our training set of metal oxides to include non-titanates: Hf- and Zr-based perovskites and cubic double perovskites, to explore local strain sensitivity in other materials, as well as other high permittivity mechanisms besides the strain tuning of Ti–O interactions. We train and test the two classes of models using the dataset, describe their performances, and discuss their similarities and differences.

## Dataset

### Derivative structure enumeration

**Figure 1:**
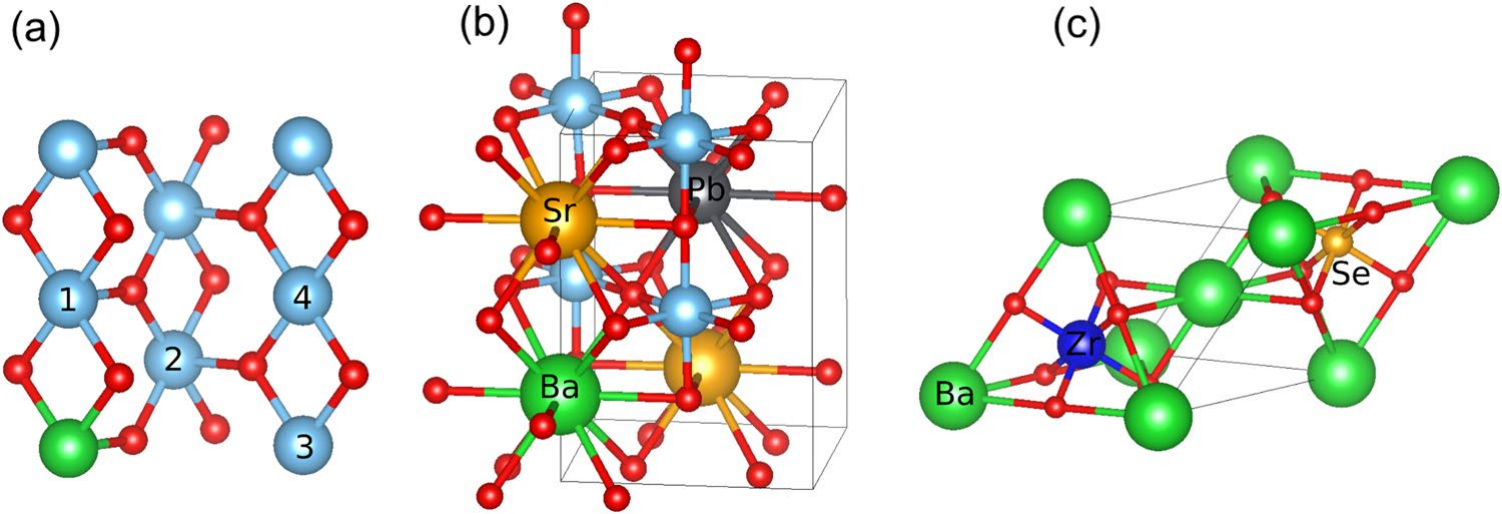
(**a**) Co-doping motifs in rutile. Numbers show different positions of the second atom in the co-doping pair with respect to the first (central) atom, shown in green; (**b**) BaPbSr_2_Ti_4_O_12_ structure derived by substitutions in the Pnma CaTiO_3_; (**c**) Ba_2_SeZrO_6_ cubic double perovskite with a rocksalt B-site cation ordering.

For ML model training, we generate an in-house ab initio dataset with optimized geometries and static electronic and ionic dielectric tensors of candidate large dielectric constants materials. Oxides composed of alkaline earth and transition metal elements, with rutile, perovskite, Ruddlesden-Popper, and orthorhombic Cmcm structures, were used as prototypes for co-doping and isovalent substitutions. Co-doping in rutile TiO_2_ and rutile phases^21^ of SiO_2_ (stishovite) and SnO_2_ was explored for boosting the dielectric permittivity in these materials^7,9,13,22^. In rutile prototypes, aliovalent co-doping with III-V (Al^3+^, Ga^3+^, In^3+^, Sc^3+^, Y^3+^, La^3+^–V^5+^, Nb^5+^, Ta^5+^) and II-VI (Mg^2+^, Ca^2+^, Sr^2+^, Ba^2+^–Cr^6+^, Mo^6+^, W^6+^) ions was performed at the X = Ti^4+^/Si^4+^/Sn^4+^ cation sites, with the general formula A^(4-δ)+^B^(4+δ)+^X_2_O_8_, with δ = 1,2. Here the ionic valencies were presumably assigned; however, the charge transfer may result in zero band-gap metallic states after self-consistent electronic structure calculations. Such metallic states were excluded in the present study. The co-doping motifs are shown in Fig. 1a. The prototypes for isovalent substitutions were Pnma perovskite CaTiO_3_, Ruddlesden-Popper phases^23,24^ Sr_2_TiO_4_ and Sr_3_Ti_2_O_7_, cubic perovskite barium zirconate^25^ BaZrO_3_, and Cmcm strontium zirconate^26^ SrZrO_3_. Ca^2+^, Sr^2+^, Ba^2+^, and Pb^2+^ isovalent substitutions were performed on the alkaline earth metal site and Ti^4+^, Zr^4+^, and Hf^4+^ on the transition metal site. An example BaPbSr_2_Ti_4_O_12_ structure, obtained by the Ba, Pb, Sr substitutions on the A-site of the Pnma CaTiO_3_, is shown in Fig. 1b. Finally, substitutions in double perovskites, with a general formula A_2_B’B’’O_6_, were performed. We employed the formula A^2^^+^_2_B’^(4-δ)+^B’’^(4+δ)+^O_6_, with A = Ca^2+^, Sr^2+^, Ba^2+^, and δ = 1, 2, 3, constraining the charge states of B’ and B’’ to maintain neutrality. Here, high symmetry cubic structures were explored, Fig. 1c, with B’ and B’’ ions selected from across the periodic table.

Symmetry unique derivative supercell structures^27,28^ were generated by substitutions on the sites of the primitive cell lattice, as implemented in the ICET code^29^. The properties of 6808 structures with isovalent substitutions and 453 co-doped structures were calculated. The structures were first optimized, and then DFPT response was obtained. Out of 6808 substituted structures, 1991 structures were dynamically stable, and 4817 had imaginary phonon frequencies. In co-doped rutiles, 159 structures were dynamically stable, and remaining 294 structures unstable. Relaxations along the displacements of the unstable phonon modes were not pursued, and the dynamically unstable structures were excluded from regression model training. The dataset consisting of the 1991 substituted and 159 co-doped dynamically stable structures was used in both classification and regression training.

For the above dataset, we evaluated the optimized geometry, Born charges, phonon vibrational modes, and dielectric permittivity by using density functional theory and density functional perturbation theory (DFPT)^30^ implemented in the VASP package^31^. The calculated data is available and top 20 materials which have large dielectric permittivity are listed in Table S1.

## Predicting the dielectric constant and dynamic stability with random forest

In this section we employed the RF machine learning method. First, we constructed machine learning regression models to predict the dielectric constant. Because the enhancement of dielectric permittivity is closely related to softening of the optical phonon modes^32,33^, we made another machine model, namely, a classification model in terms of the minimum optical mode. One of the features of RF is accessibility of importance of descriptors as will be discussed in detail.

The RF machine learning method employs the bootstrap aggregating for sampling the decision/prediction trees to improve the stability and accuracy, and shows reasonably good performance in most cases for classification and regression. The input variables included elemental properties and structural features encoded by using the pymatgen^34^ and matminer^35^ packages. The list of the features used is given in Table S2 of the Supplementary Information; the choice of the 45 descriptors was similar to that in a previous study^19^.

The calculated dielectric constants for the given structures in the dataset were used to train the RF regression^36^, as implemented in the scikit-learn^37^ code. The ionic and electronic contributions were treated separately, and the decimal logarithm of the dielectric constant was taken as the target variable for the model. The logarithm value was used to mitigate the disproportional effect of the systems with large dielectric constants on the model. Tables S3–S5 provide the results of the hyperparameter space search. The number of decision trees was set to 150, and the maximum tree depth was not constrained, by keeping the minimum number of samples required to split internal nodes at the default value of 2. In evaluation, fourfold cross-validation was used. RF is robust against the variation of hyperparameters, with the default hyperparameters values resulting in nearly same performance as those obtained by search using grid or Bayesian optimizations. The root-mean-squared errors (RMSE) and coefficients of determination (*R*^2^) were used as the metrics for model performance.

**Figure 2:**
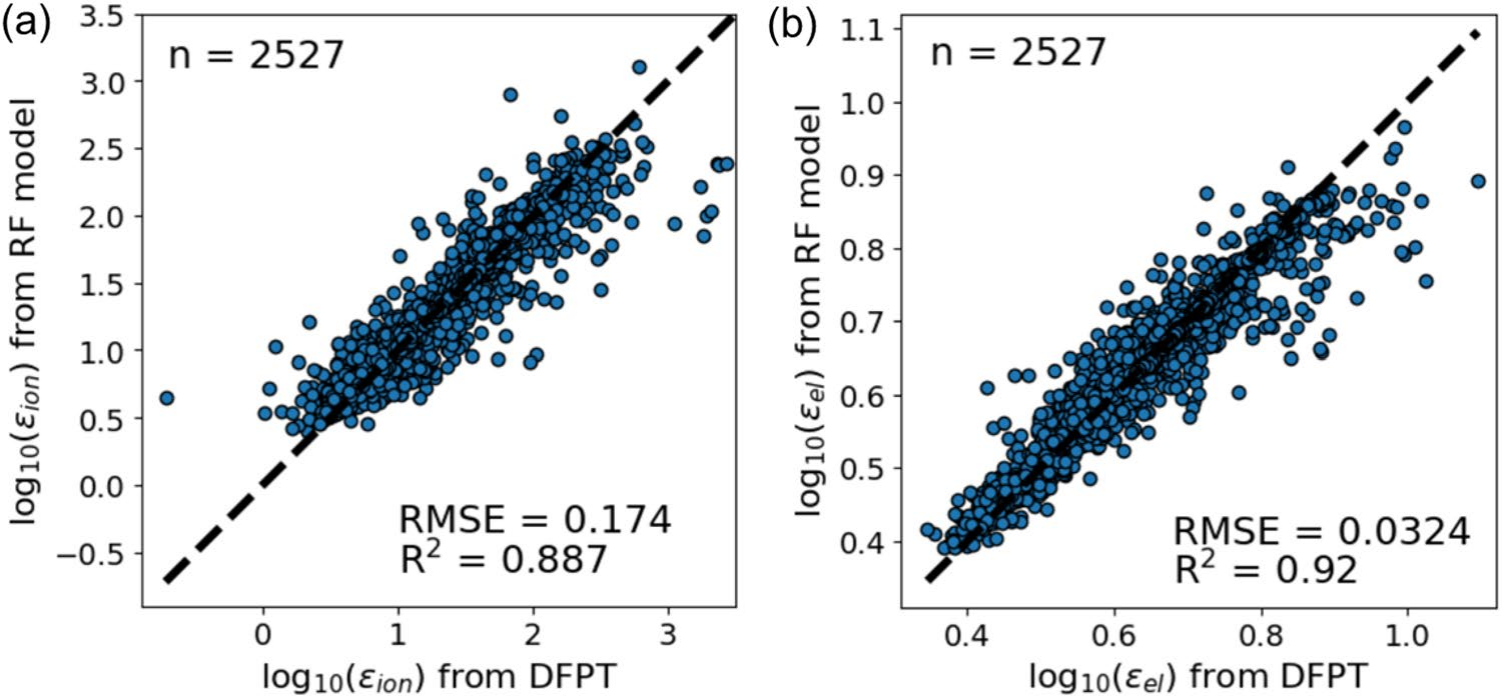
Cross-validation parity plot for the calculated and RF-predicted dielectric constant: (**a**) ionic contribution and (**b**) electronic contribution.

The parity plots for the calculated and RF predicted dielectric constant are shown in Fig. 2. The calculated permittivities vary in a wide range and noticeably reach *ɛ*_ion_ > 100. The large dielectric permittivities are obtained with doped and substituted titania as previously reported^8^. The electronic contribution is reasonably small, *ɛ*_el_ > 10, as in the intrinsic paraelectric materials. Thus, a large dielectric permittivity can be achieved by realizing a large *ɛ*_ion_. After optimizing the hyperparameters of the RF regression model as described above, the RMSE = 0.174 and *R*^2^ = 0.887 for the ionic permittivity, and RMSE = 0.032 and *R*^2^ = 0.921 for the electronic permittivity were obtained for the test data in the cross-validation. These values are better than the previous work using the RF model constructed for the metal oxides in the Materials Project^20^, RMSE = 0.148 and *R*^2^ = 0.73 for the ionic permittivity^19^. It is important to note that our model successfully predicts the dielectric constant even with a significant structural distortion by doping and the corresponding quite high permittivity.

We also created a classification model using the phonon frequency of the smallest optical mode as the supervised data. All materials with a band gap greater than 0.2 eV, including those with imaginary phonon frequencies, were used. A classification model was generated by labeling three classes of phonon frequencies *ω*: *ω*_opt_^2^ ≤ 0, 0 < *ω*_opt_ ≤ 2 THz, and *ω*_opt_ > 2 THz. The first class, *ω*_opt_^2^ ≤ 0, indicates dynamical instability. The instability may include a phase transition to a ferroelectric phase. The second class, 0 < *ω*_opt_ ≤ 2 THz, is of interest in terms of possibility of colossal dielectric permittivity, as it is inversely proportional to *ω*_opt_^2^^30,32,38^. The third class, *ω*_opt_ > 2 THz, is categorized into normal paraelectric materials. For the classification model, we used the RF classification of the scikit-learn code. The same descriptors and the optimization procedure were used as in the regression model.

**Figure 3:**
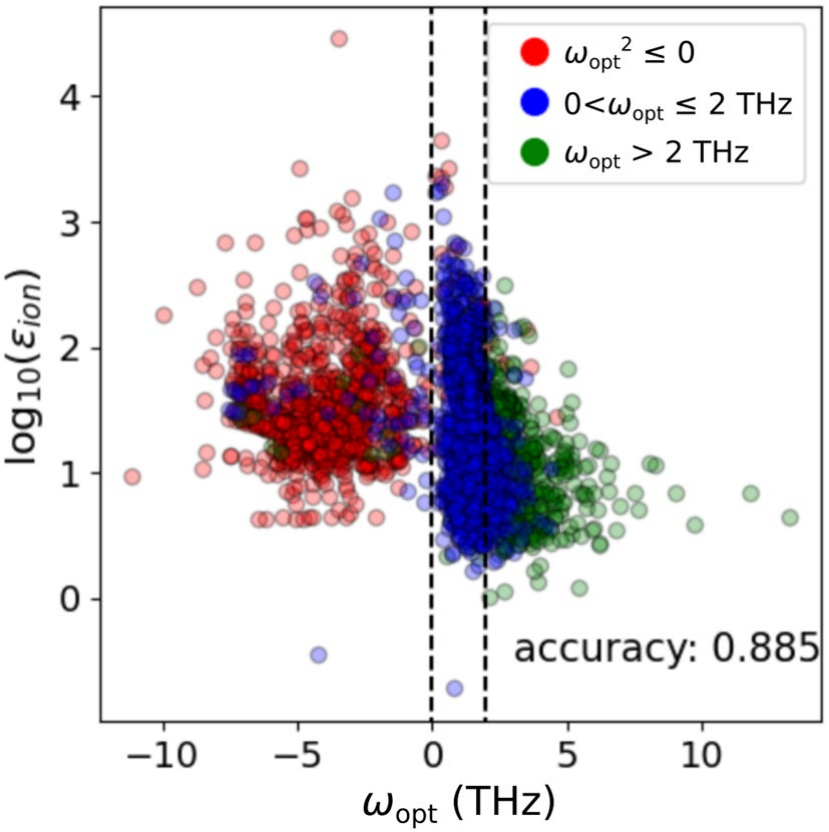
Distribution of ionic contribution to the frequency and permittivity of the smallest optical mode. Colors are the predicted results of random forest classification. Red circles indicate that the model predicted *ω*_opt_^2^ ≤ 0, blue circles indicate 0 < *ω*_opt_ ≤ 2 THz, and green circles indicate *ω*_opt_ > 2 THz.

Prediction results of the classification model are shown in Fig. 3. Note that the imaginary part of the phonon frequency was expressed as a negative number in the plot. Regarding the prediction accuracy, the accuracy and *F*_1_ scores were 0.885 and 0.880, respectively.

**Figure 4:**
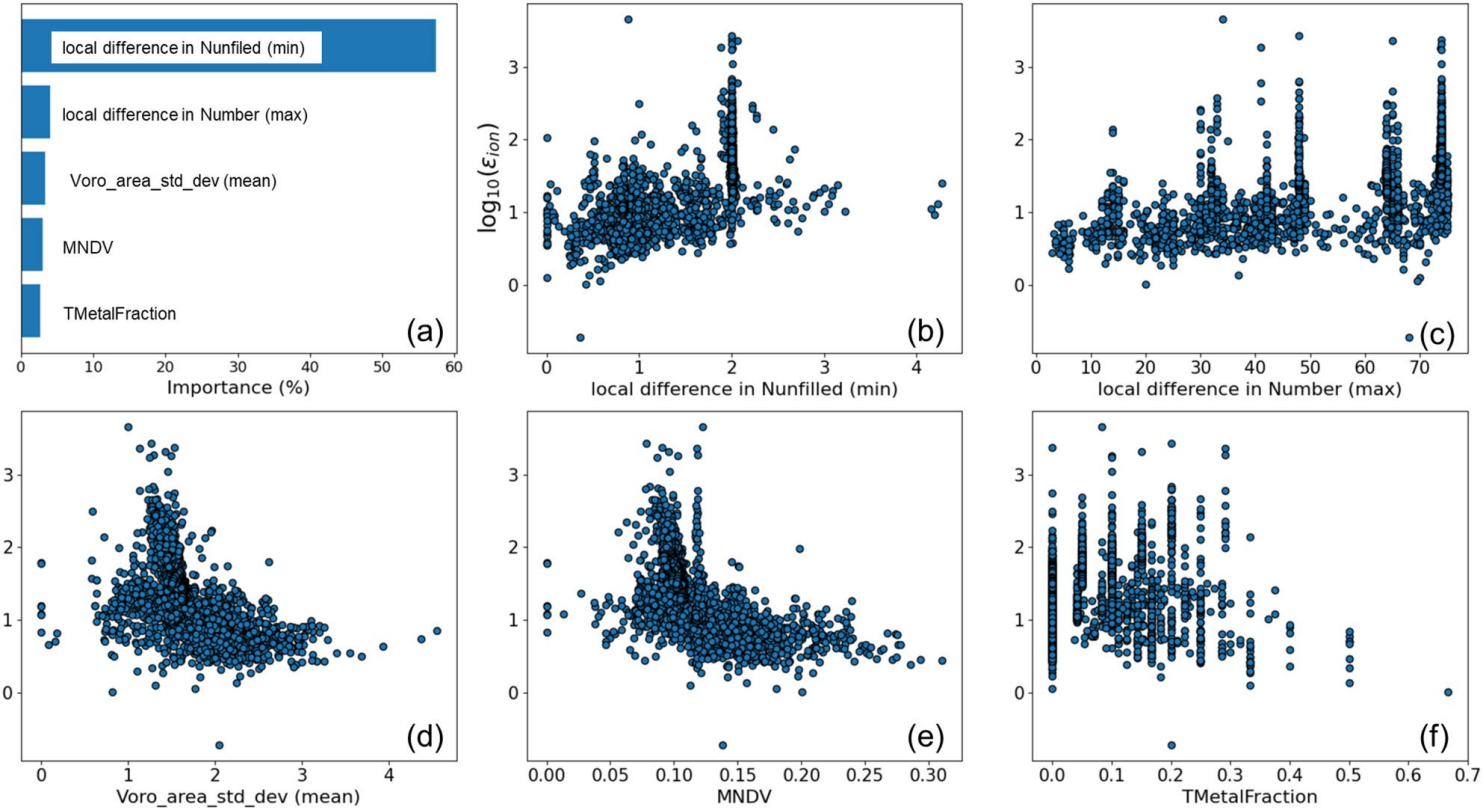
(**a**) Importance values of the five most important descriptors of the RF regression models of the ionic dielectric constants *ε*_ion_. (b)-(f) Distributions of the ionic dielectric constant with respect to: (**b**) local difference in Nunfilled (min), (**c**) local difference in Number (max), (**d**) Voro_area_std_dev (mean), (**e**) mean neighbor distance variation, and (**f**) transition metal fraction descriptors.

Because the above machine learning models predict the dielectric constant with a reasonable accuracy, it is interesting to investigate which descriptors are important for the prediction. The importance of descriptors in the RF regression of ionic permittivity and their correlation coefficients are shown in Fig. 4a. We observe that local differences in atomic properties and structures turn out to be important. For instance, the most important descriptor for the regression is the minimum value of the absolute local differences between the number of unfilled electrons in each atom and its neighbors (local difference in Nunfilled (min)) defined as min_*i*_(*δ*(*N*_unfilled, *i*_)). The local difference for each atom or site *i* is calculated using the following equation^39^:

$$\delta \left({p}_{i}\right)= \frac{{\sum }_{n}{A}_{ni}\left|{p}_{n}-{p}_{i}\right|}{{\sum }_{n}{A}_{ni}}.$$

Here, the sum is taken over all nearest neighbor sites *n* as determined by the Voronoi tessellation, *p*_*n*_ is the property (e.g. atomic number) of site *n*, and *A*_*ni*_ is a weight that corresponds to the area of the facet on the tessellation corresponding to that neighbor. The second most important descriptor is the maximum of the differences in the atomic numbers *Z* among neighbors (local difference in Number (max)) defined as max_*i*_(*δ*(*Z*_*i*_)). The third most important descriptor is the mean of the standard deviations of the Voronoi areas around each atom (Voro_area_std_dev (mean)). The fourth most important descriptor is the mean of the neighbor distance variation, *MNDV*, which indicates the degree to which the atoms are displaced from the high-symmetry positions, and the degree to which the lattice is distorted with respect to the high-symmetry structure, and is calculated from the following equation^19,39^:

$$MNDV=\frac{1}{{N}_{\rm atom}}{\sum }_{i}\frac{{\sum }_{n}{A}_{ni}|{r}_{ni}-{\bar r}_{i}|}{{\bar r}_{i}{\sum }_{n}{A}_{ni}}.$$

Here, *N*_atom_ is the number of atoms in the unit cell, and $\bar{r}_i$ is the average nearest neighbor distance for atom *i*, i.e. the sum over *n* of *r*_*ni*_, distances between atoms *i* and *n*, weighted by *A*_*ni*_/Σ_*n*_*A*_*ni*_. The fifth important descriptor is the transition metal fraction in the material (TMetalFraction)^40^, the ratio of the number of transition metal atoms to the total number of atoms in the unit cell. This descriptor does not require any structural information, and is based on the compositional information only. In Figs. 4b–f, we plot correlations of some important descriptors with the ionic dielectric constant. There is a high correlation coefficient of “local difference in Nunfilled (min)” with *ɛ*_ion_; however, most of the high *ɛ*_ion_ oxides are found around the intermediate descriptor value ~ 2. Around this value, there are many co-doped materials with both large and small *ɛ*_ion_ due to variation of their local configurations. We found similar correlations between *ε*_ion_ and both “Voro_area_std_dev (mean)” and *MNDV*. Both of these descriptors are related to geometrical symmetry, with smaller values indicating higher symmetry. These results suggest that just the right amount of asymmetry is needed for permittivity boosting. The trend is consistent with previous studies^19^, and our finding of the optimal value of the max(*d*_Ti-O_) descriptor^8^. It was also reported that the Born effective charge of BaTiO_3_ decreases with displacement from the cubic symmetry^41^ and that some polycrystalline perovskite oxides tend to have larger dielectric constants with increasing symmetry in the DFPT calculations^42^. The fact that such a trend was also observed in co-doping suggests that co-doping may control the symmetry of the local structure and increase the dielectric constant.

**Figure 5:**
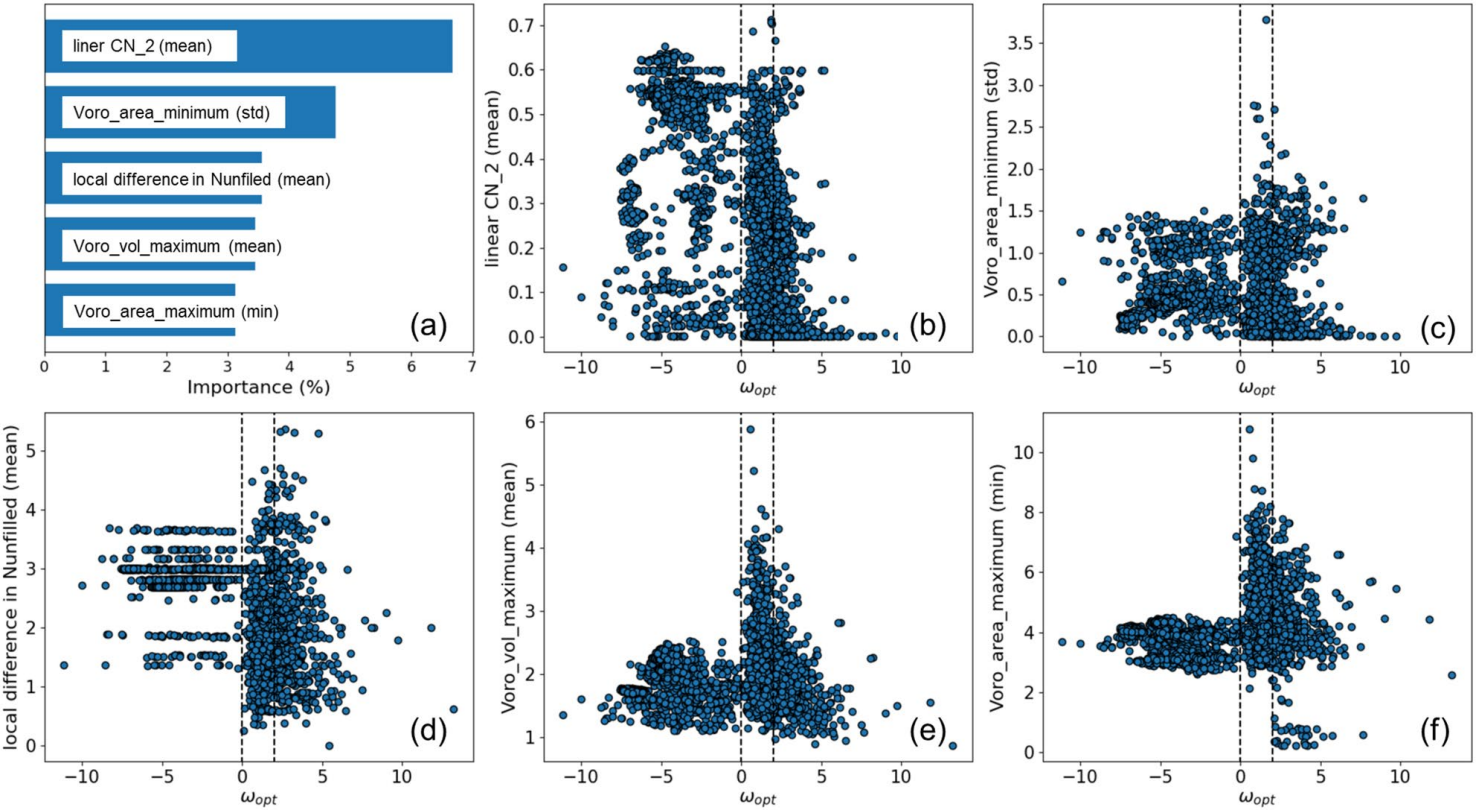
(**a**) Importance values of the five most important descriptors of the RF classification model for the frequency of the lowest optical mode. (**b**–**f**) Distribution of the lowest optical mode frequency with respect to: (**b**) linear CN_2 (mean), (**c**) Voro_area_minumum (std), (**d**) local difference in Nunfilled (mean), (**e**) Voro_vol_maximum (mean), and (**f**) Voro_area_maximum (min) descriptors.

The importance of descriptors in the RF classification for phonon frequencies was also investigated. The descriptor importance for the RF classification of phonon frequencies is shown in Fig. 5a, and their correlations with the frequency in Fig. 5b–f. The most important descriptor is the mean of the linear coordination number, (linear CN_2(mean)), indicating how similar the atomic environment is to that of a linearly 2-coordinated atom. Coordination numbers are order parameters assuming values between 0 and 1 quantifying coordination patterns of atoms^43^. Similarity to 2-coordinated linear geometry may reflect chain instability^44,45^ driving ferroelectric transitions in some oxides. Three of the remaining top descriptors are related to the shape of the Voronoi partitions, while another is min_*i*_(*δ*(*N*_unfilled, *i*_)), so that all of the 5 top descriptors are related to the local structure. There is an unusual dispersion in the plot of the minimum phonon frequency and the average value of Voronoi volume maxima around each atom (Voro_vol_maximum (mean)), as shown in Fig. 5e. Here we show that most of the samples have 0 < *ω*_opt_ ≤ 2 THz when this descriptor takes more than three. It is suggested that controlling the local structure may reduce the phonon frequency and increase the dielectric constant.

**Figure 6:**
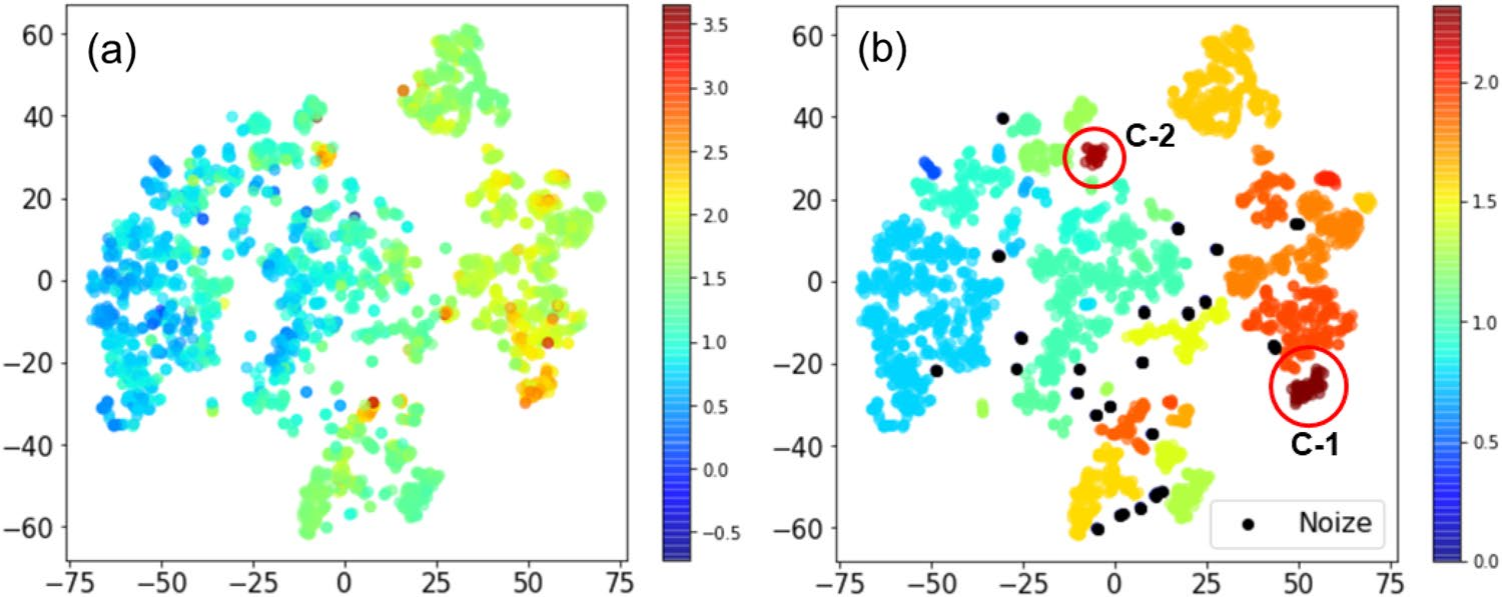
(**a**) two-dimensional descriptor space evaluated by t-SNE. The color scale indicates logarithmic dielectric constant of the material; (**b**) the results of clustering using DBSCAN where the averaged logarithmic dielectric constant in each cluster is shown in the color scale. The two highest clusters at red circles are named C-1 and C-2 for the analysis in Fig. 7.

**Figure 7:**
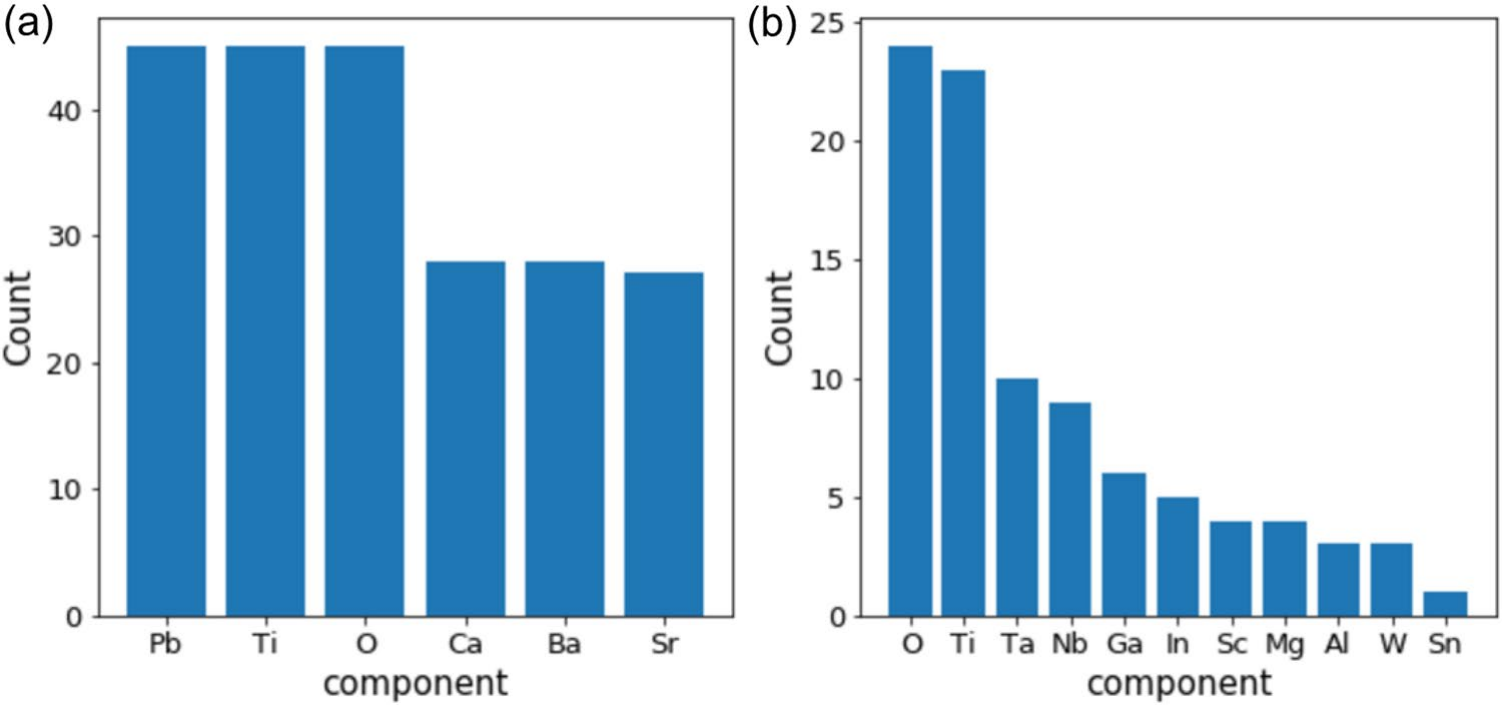
Counts of the elements of which the materials included in the two clusters, C-1 (**a**) and C-2 (**b**) indicated in Fig. 6b, are composed.

Figure 6a visualizes the data distribution in a two-dimensional descriptor space. The t-distributed stochastic neighbor embedding method (t-SNE)^46^ was used to reduce from 45-dimensional vectors of matminer descriptors into two dimensions. The distances among the data points represent similarity of the descriptor vectors. We observed clustering of the data points where each cluster has overall similar dielectric constant. This means that the descriptors used properly express the magnitude of the dielectric constant. In order to make clustering in the descriptor space, we employed the density-based spatial clustering of applications with noise (DBSCAN)^47^. The DBSCAN is a density-based clustering algorithm. Different from the *k*-means method, DBSCAN does not require to specify the number of clusters in advance. The data points which do not belong to any clusters are regarded as noise. With DBSCAN, we were able to divide the data set into 40 clusters (Fig. 6b). The average of the dielectric constant in each cluster is clearly distinct. For the two clusters, C-1 and C-2, which show the highest dielectric constant, we counted appearance of the elements in composition of the materials included in each cluster as shown in Fig. 7. The C-1 cluster consists of Pb and alkaline earth metals indicating doping systems for perovskite CaTiO_3_. On the other hand, the C-2 cluster mainly includes co-doped rutile TiO_2_. Both types of modification turn out to be effective for boosting the dielectric permittivity.

## Graph convolutional neural networks

We also utilized graph convolutional neural networks (GCNNs) with SchNet^18^ architecture for the regression and classification tasks. GCNNs^48,49^ are well suited for learning properties of molecules and crystals^17,50–52^, whose structure can be naturally represented by graphs. The target property is learned through local message passing^53^ among the neighboring nodes. Crystal GCNNs operate with the node (atom) and edge (bond) attributes of the graph representing the spatial connectivity of atoms in a crystal^17^. In SchNet, atomic numbers are used for initial node embeddings, while a trainable edge filter **W**(*r*): ℝ^+^ → ℝ^*m*^ is created by passing the Gaussian expansions of interatomic distances *r* through a multilayer perceptron. Here, *m* is the number of edge filter attributes. The convolution^18^ on each node *i* is a sum of element-wise products over all neighbors *j* of node *i*, i.e. $\Sigma_{j \in \mathcal{N}(i)}$ **x**_*j*_⊙**W**(|**r**_*j*_-**r**_*i*_|). The complete message passing/interaction layer has additional perceptrons before and after the convolution, and after several interactions, node attributes are pooled into the target value.

**Figure 8:**
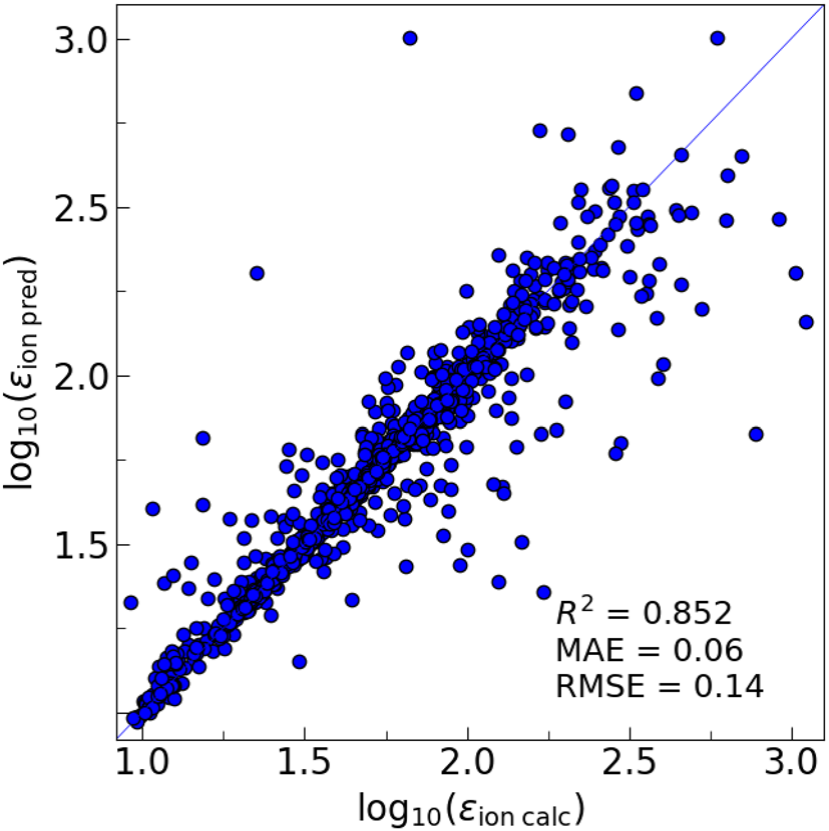
Cross-validation parity plot for the DFPT-calculated and SchNet GCNN-predicted ionic dielectric constant.

Figure 8 shows the parity plot for a fivefold cross-validation SchNet training with log_10_(ε_ion_), yielding the coefficients of determination *R*^2^ = 0.852, and RMSE = 0.14. The performance is somewhat better than that of RF, with *R*^2^ = 0.887 and RMSE = 0.174. Here, log_10_(ε_ion_) is taken as the target value, as using the actual ε_ion_ for the target value results in large absolute errors, Fig. S1. We also compared the performance of SchNet and CGCNN^17^, consistently obtaining better results with SchNet. SchNet was found to be rather robust against hyperparameter variation, with rather wide regions of stable performance, as seen in Fig. S2. In particular, 3 interaction layers were sufficient to achieve close to optimal performance.

We benchmarked the improvement of the performance of SchNet and CGCNN with the size of the training dataset. The dataset with dynamically stable structures was split at different ratios, using one part for training, and the remaining part for validation. Both SchNet and CGCNN show significant improvements with increasing dataset size, as seen in Fig. S3, with SchNet performing better than CGCNN. The overall SchNet performance shows a large benefit of using a simple network architecture for high quality prediction of the dielectric constant of complex materials.

**Figure 9:**
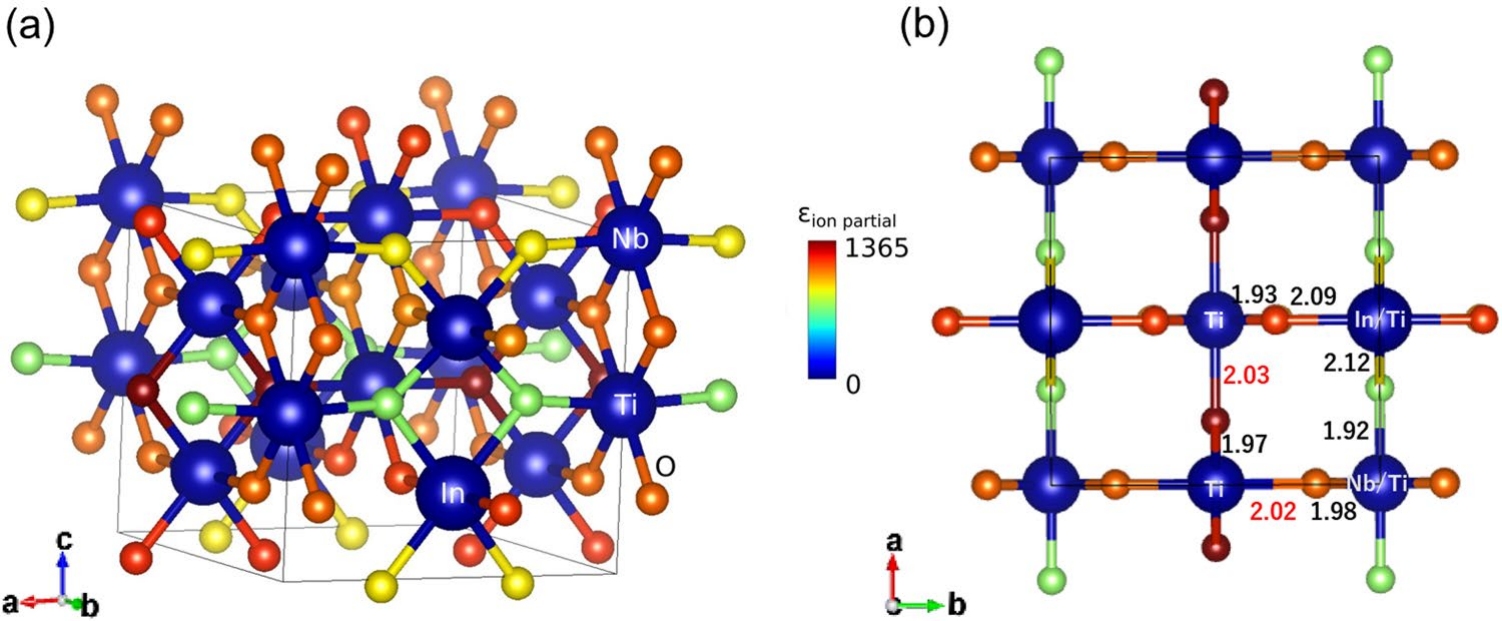
Atomic (node) contributions to dielectric constant with "heatmap" atom coloring as defined in the color bar, evaluated for In-Nb co-doped rutile TiO_2_ by the trained SchNet model, (**a**)—side view, (**b**)—top view. The bond lengths are shown in (**b**) highlighting in red an optimal Ti–O bond length (about 2.02 Å) to achieve a large ε_ion_ as presented in Ref.^8^.

**Figure 10:**
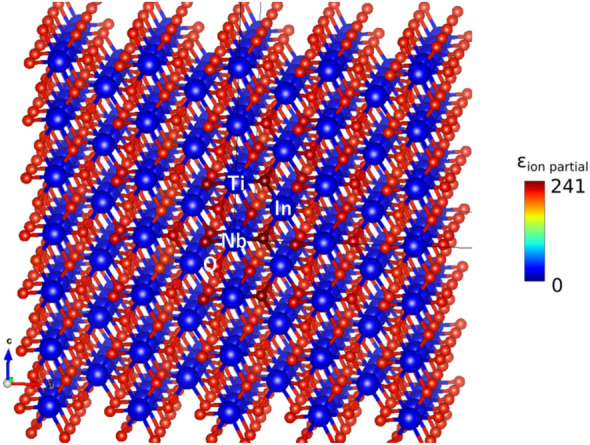
Atomic (node) contributions to dielectric constant with "heatmap" atom coloring as defined in the color bar, evaluated for an unrelaxed large unit cell of In-Nb co-doped rutile TiO_2_ by the trained SchNet model.

One can obtain spatial information about the dielectric constant contribution by querying the node (atom) attributes in the last interaction layer before average pooling. In Fig. 9, individual atomic contributions in In-Nb co-doped rutile TiO_2_, which shows one of the largest dielectric constants in the dataset, are shown. The GCNN assigns the largest contribution to O atoms, while Ti is assigned the smallest contribution. This is probably because the GCNN can noticeably detect a difference in local configuration around O atom to assign a wide range of dielectric constants. Significantly large dielectric contributions are seen at O atoms whose Ti–O bond lengths are around 2.02 Å as shown in Fig. 9b. The observation is indeed consistent with the results obtained for doped TiO_2_ and pristine rutile TiO_2_, in which the softening of Ti–O mode occurs at max(*d*_Ti-O_) ≃ 2.02 Å as the result of the strain induced by doping^8^. This unique feature of the trained GCNN model gives visualization of the atomic contributions to dielectric constant in any unit cell. Figure 10 shows the atomic contributions to dielectric constant in an unrelaxed large rutile TiO_2_ unit cell including a pair of In-Nb co-doping. While the local strain is not introduced because each atomic position is not optimized, relatively large contributions around the In-Nb co-doping site are predicted by the trained SchNet model. This is interpreted as contributions to dielectric constant from chemical coordination without changing bond lengths. One can expect additional boosting of dielectric constant in the fully relaxed structure where local stain around doping is introduced. Further performance improvements can be achieved with models utilizing not only information about bond distances, but also bond angles^54^.

A computationally efficient workflow must utilize ML-only steps. Thus, additional ML models for structural optimization or band gap prediction are needed for a closed-cycle screening and discovery of dielectric materials, and our dielectric constant models, which were trained with optimized geometries, must be coupled with another ML model for structural relaxations. Alternatively, one could train a dielectric constant model directly with unrelaxed structures. The comparison, evaluation, benchmarking, and analysis of such models trained with relaxed vs. unrelaxed geometries has not been performed here and is deferred to future work. We also trained a SchNet GCNN using DFT electronic band gaps as target values and relaxed geometries as inputs. The model shows rather good performance, as seen from the parity plot, Fig. S4, and can be used for screening for dielectrics with large gaps. Eventually, it is desirable to search for completely new structures beyond the derivatives by doping. While proposing new structures including their synthesizability with ML-only approaches is still quite challenging, we believe that our prediction models hold promise for use in new materials discovery.

In conclusion, we carried out ML modeling to predict the dielectric constants of oxides and classify their dynamic stability based on the structural information. Two classes of ML models, random forest and graph convolutional neural networks, are used for classification and regression. Both models show similar cross-validation performance with the coefficient of determination R^2^ ~ 0.8–0.9 for predicting the dielectric constants in a wide range 10–10^3^. Feature importance analysis shows that the local differences of atomic and geometric features play a large role in determining the value of the dielectric constant of the material. Both approaches show fast performance and are suitable for high throughput screening and evaluation of high dielectric constant materials.

## Methods

The ionic dielectric response was calculated using a density functional perturbation theory (DFPT) approach^30^, as implemented in the VASP package^31^. PBEsol functional^55^ with Hubbard *U* correction^56^ was used. *U* = 3 eV was applied to *d* electrons in transition metals, as employed in previous work^19^, except for Ti and Sc, where *U* = 0 was used^14^, and *U* = 5 eV was applied to *f* electrons in rare earths. Table S6 compares the calculated values of the dielectric constant obtained with different *U* values with experimental ones, showing *U* = 3 eV to be a reasonable choice except for the titanates and scandium oxide, where *U* = 0 is the best choice. Projector-augmented wave (PAW) method was used to treat core electrons. Recommended PAW potentials were employed for all elements except Ti, where valence 4 “Ti” potential was found to better represent the vibrational and optical properties of titanium dioxide^14^. The k point grid density of 3000 k points·atom (approximately corresponding to a distance of 0.2 Å^−1^ between the k points) was used for the integration over the Brillouin zone. The cell shape and atomic position were fully optimized prior to DFPT calculations. The norms of all forces acting on atoms were minimized to within 0.005 eV/Å. We employed this criterion for computational efficiency, although we observed that it led to errors in some cases, in particular for the dynamically unstable structures.

The ionic part of the dielectric tensor ε_ion *αβ*_ is obtained from DFPT as a sum over phonon modes, according to^30,38,57^

1 $${\varepsilon }_{\rm{ion} \,\alpha \beta }=\frac{{e}^{2}}{{{\varepsilon }_{0}M}_{0}V}{\sum }_{\nu \,}\frac{{\bar{Z}*}_{\nu \alpha }{\bar{Z}*}_{\nu \beta }}{{\omega }_{\nu }^{2}}.$$

Here, *e* is an elementary charge, *M*_0_ is a mass reference, *V* a unit cell volume, ${\bar{Z}*}_{\nu \alpha }={\sum }_{\kappa \beta \,}{Z*}_{\kappa ,\alpha \beta }{\left({M}_{0}/{M}_{\kappa }\right)}^{1/2}{\xi }_{\nu ,\kappa \beta }$ is the *α*th Cartesian component of the unnormalized effective charge vector for phonon mode *ν*, and *ω*_*ν*_ is mode frequency. *ξ*_*ν*,*κβ*_ are the eigenvectors of the dynamical matrix, normalized according to Σ_*κβ*_*ξ*_*ν*,*κβ*_* ξ*_*ν’*,*κβ*_ = *δ*_*νν’*_, and Z*_κ,*αβ*_ are the atomic Born charges. Note that only modes with nonzero effective charges contribute to the static tensor Eq. (1). The interatomic force constants are used to construct the dynamical matrix. The phonon frequencies were obtained by diagonalizing the dynamical matrix. The mode must be polar, which also makes it a candidate for ferroelectric transition when soft. Note that the large ionic epsilon can be achieved by either increasing *Z* or decreasing *ω*. The latter means softening of the phonon mode, which leads to a ferroelectric instability.

The importance *I*(*j*) of feature *j* in a random forest in scikit-learn is defined using Gini importance as

$$I\left(j\right)={\sum }_{i=1}^{n\in F\left(j\right)}\left({w}_{i}{C}_{i}-{w}_{left\left(i\right)}{C}_{left\left(i\right)}-{w}_{right\left(i\right)}{C}_{right\left(i\right)}\right),$$

where* F*(*j*) is the importance of feature *j*, *w*_*j*_ is a weighted number of samples reaching node *i*, and *C*_*i*_ is an impurity value of node *i*. Left(*i*) and right(*i*) are the right and left child nodes on node *i*, respectively.

The impurity value *C* in the classification problem is

$$C\left(i\right)= {\sum }_{k=1}^{N}{f}_{k}(1-{f}_{k}),$$

where *N* is the number of unique labels and *f*_*k*_ is the frequency of label *k*.

For regression problems, the impurity value *C* is

$$C\left(i\right)=\frac{1}{M}{\sum }_{k=1}^{M}{\left({y}_{k} -\mu \right)}^{2},$$

where *M* is the number of instances, *y*_*k*_ is label for an instance and *μ* is the mean value given by $\frac{1}{M}{\sum }_{k=1}^{M}{y}_{k}^{2}$. The final value is output after normalizing the Gini importance of each feature.

### Supplementary Information

Supplementary Information.

## Data Availability

The datasets generated during the current study will be available at 10.17632/m5jhkc3p9d.1 and the other data used in the study will be available on reasonable request from the corresponding author.
