# Supplementary material for: Machine learning and atomistic origin of high dielectric permittivity in oxides
Source: Sci Rep. 2023 Dec 14;13:22236. doi: 10.1038/s41598-023-49603-2 (PMC10721917; doi:10.1038/s41598-023-49603-2)
Supplement: Supplementary file 1 — Supplementary Information. [file 41598_2023_49603_MOESM1_ESM.pdf]

Supplementary Information for: “Machine learning and atomistic origin of high dielectric permittivity in oxides”

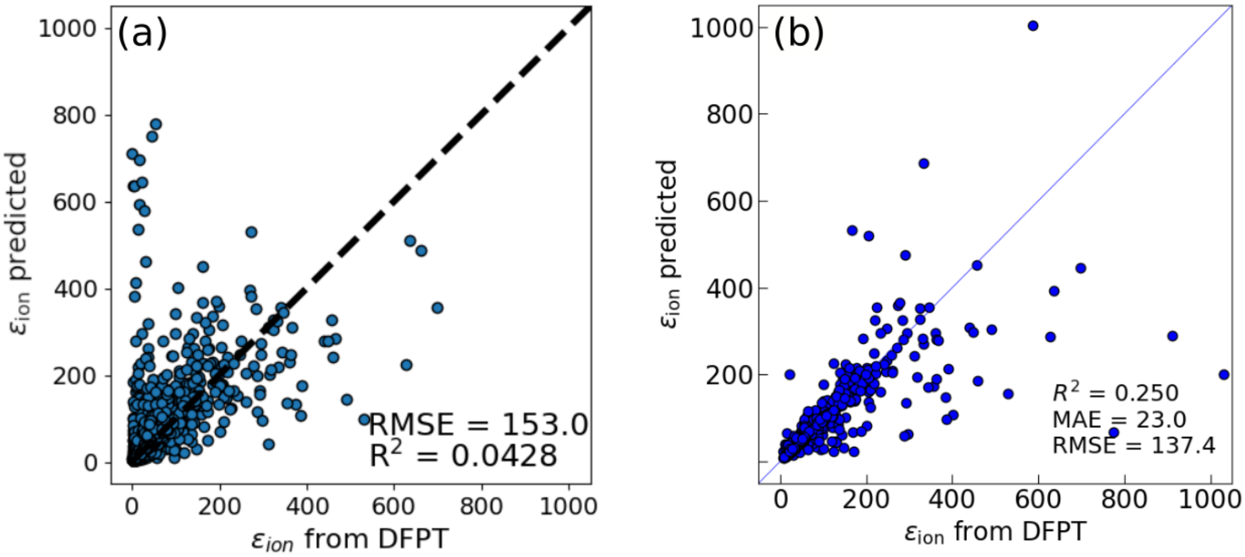

Figure S1. Parity plots for (a) random forest and (b) SchNet graph convolutional neural network models for ionic dielectric constant.

Hyperparameter tuning in SchNet GCNN.
To investigate the hyperparameter dependence of the GNN model with the created SchNet, we further optimized the hyperparameters. The results are shown in Figure S2. num_layer is the number of interaction layers, max_radius is the maximum edge length, and edge_attr_size is the number of edge features. The results show that SchNet is relatively robust to the number of layers and filters, and that edge length and number of features have a greater impact on accuracy.

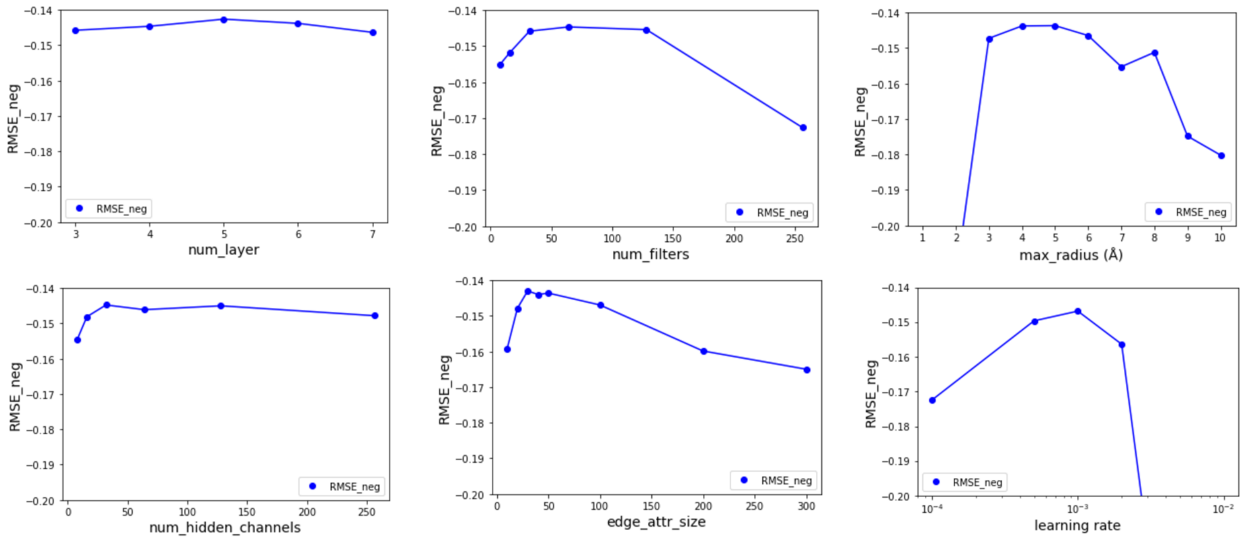

Figure S2. Results of SchNet hyperparameter search, showing the negative of RMSE vs. hyperparameter value, with larger values indicating better model performance. Number of interaction layers (num_layer), number of edge filter attributes *m* (num_filters), graph cutoff radius (max_radius), node feature embedding size (num_hidden_channels), number of Gaussian functions in the edge distance expansion (edge_attr_size), and initial learning rate were tested.

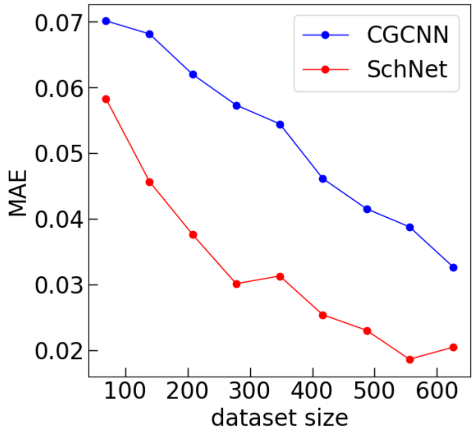

Figure S3. Performance improvement of SchNet and CGCNN with the size of the training dataset.

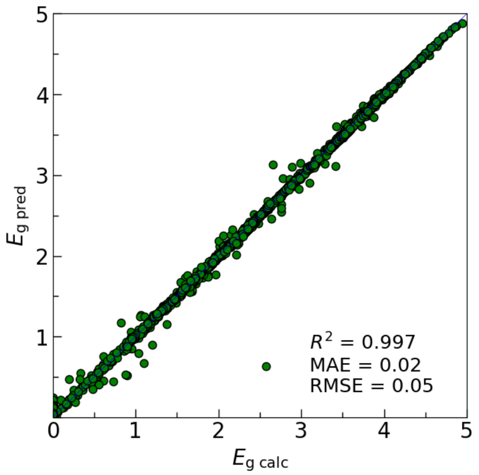

Figure S4. Parity plot for electronic band gap prediction with a SchNet model trained with DFT band gaps and relaxed geometries as inputs.

Table S1. Top 20 materials that have large dielectric permittivity.

Formula	$<\varepsilon_{ion}>_{harm}$	$<\varepsilon>$
$Sr_2Ba_2Ti_4O_{12}$	752	2674
$Sr_2BaPbTi_4O_{12}$	681	698
$Ca_2BaZrPbTi_3O_{12}$	647	2068
$Sr_3BaTi_4O_{12}$	622	636
$Ca_2SrBaTi_4O_{12}$	597	628
$Ca_2SrZrHfPbTi_2O_{12}$	533	1815
$Sr_2BaPbTi_4O_{12}$	427	456
$Ca_4Sr_2Ba_2Ti_8O_{24}$	424	463
$InNbTi_6O_{16}$	418	587
$Ba_2Zr_4Pb_2O_{12}$	411	556
$Ba_3PbTi_4O_{12}$	401	448
$Ca_5Sr_2BaTi_8O_{24}$	399	643

$Ca_6Ba_2Ti_8O_{24}$	361	398
$InTaTi_6O_{16}$	350	912
$Ca_2Sr_4Ba_2Ti_8O_{24}$	341	452
$CaBaZrHfPb_2Ti_2O_{12}$	324	1110
$Sr_3PbTi_4O_{12}$	322	324
$SrBa_2PbTi_4O_{12}$	319	324
$SrBaPb_2Ti_4O_{12}$	318	333
$CaSrBaPbTi_4O_{12}$	315	439

Table S2. List of descriptors for the random forest model.

Descriptor Name
PymatgenData std_dev row
PymatgenData mean thermal_conductivity
PymatgenData std_dev melting_point
TMetalFraction
gap_AO
mass density
packing fraction
mean neighbor distance variation
avg_dev neighbor distance variation
sgl_bd CN_1 (mean)
bent 150 degrees CN_2 (mean)
linear CN_2 (mean)
trigonal planar CN_3 (mean)
pentagonal planar CN_5 (std)
octahedral CN_6 (max)
octahedral CN_6 (std)
q6 CN_12 (mean)
EwaldSiteEnergy
Symmetry_weighted_index_4 (std)
Voro_vol_maximum (mean)
Voro_area_std_dev (mean)
Voro_area_minimum(std)
Voro_area_maximum (min)
Voro_dist_std_dev (mean)
G2_80.0 (min)

G4_0.005_4.0_1.0 (std)
local difference in Number (max)
local difference in MendeleevNumber (max)
local difference in MendeleevNumber(min)
local difference in AtomicWeight (max)
local difference in AtomicWeight (mean)
local difference in MeltingT (mean)
local difference in Row (max)
local difference in Electronegativity (min)
local difference in Nvalence (std)
local difference in NsUnfilled (mean)
local difference in NdUnfilled (max)
local difference in NdUnfilled (std)
local difference in NUnfilled (max)
local difference in Nunfilled (min)
local difference in Nunfilled(mean)
local difference in Nunfilled (std)
local difference in GSvolume_pa (max)
local difference in GSvolume_pa (min)
local difference in SpaceGroupNumber (max)

To investigate the hyperparameter dependence of the random forest regression models we created, we optimized the hyperparameters. Grid Search and Optuna [1] were used for hyperparameter optimization. Optuna is a Python library for hyperparameter optimization based on Bayesian optimization. The optimization results are shown in Tables S3-S5. Both methods yielded better accuracy than the defaults, but the results were not dramatic.

Table S3. Results of hyperparameter optimization of random forests in Scikit-learn with the Grid Search and Bayesian optimization library Optuna.

	bootstrap	max_depth	max_features	min_samples _leaf	min_samples _split	n_estimators
**Default**	**True**	**None**	**Sqrt**	**1**	**2**	**100**
**Grid search**	**False**	**100**	**sqrt**	**1**	**2**	**2000**
**Optuna**	**False**	**None**	**sqrt**	**1**	**2**	**5700**

Table S4. Random Forest regression results with hyperparameters optimized by Grid Search and Optuna.

	Random Forest Default	Random Forest Grid search	Random Forest optuna
**RMSE**	**0.153236**	**0.147513**	**0.147549**
**R^2**	**0.811291**	**0.825122**	**0.825039**

Table S5. Hyperparameters of the random forests used in the optimization with Grid Search and Optuna

	bootstrap	max_depth	max_features	min_samples_leaf	min_samples_split	n_estimators
**Grid research**	**True False**	**100, None**	**Auto sqrt**	**1, 4**	**2, 10**	**500, 2000**
**Oputuna**	**True False**	**10 ~ 500 None**	**Auto sqrt**	**1~20**	**2~20**	**100~ 10000**

Table S6. Comparison of the calculated total (electronic+ionic) dielectric constants obtained with different Hubbard *U* values with experimental ones. Note PAW potential of Ti having four valence electrons, $3d^3 4s^1$, was employed [2].

Material	U=0	U=3 eV	U=5 eV	Expt.
$CaZrO_3$	39	28	25	24[(a)]
TiO_2	148	43	28	160[(b)]
$SrTiO_3$	258	51	35	300[(c)]
Sc_2O_3	17	13	11	17[(d)]

(a) Ref. [3]
(b) Ref. [4]
(c) Ref. [5]
(d) Ref. [6]

References

[1] T. Akiba, S. Sano, T. Yanase, T. Ohta, M. Koyama, Optuna: A Next-Generation Hyperparameter Optimization Framework, in: Proc. 25th ACM SIGKDD Int. Conf. Knowl. Discov. Data Min., Association for Computing Machinery, New York, NY, USA, 2019: pp. 2623–2631. https://doi.org/10.1145/3292500.3330701.

[2] P.R. Varadwaj, V.A. Dinh, Y. Morikawa, R. Asahi, Polymorphs of Titanium Dioxide: An Assessment of the Variants of Projector Augmented Wave Potential of Titanium on Their Geometric and Dielectric Properties, ACS Omega. 8 (2023) 22003–22017. https://doi.org/10.1021/acsomega.3c02038.

[3] H. Stetson, B. Schwartz, Dielectric Properties of Zirconates, J. Am. Ceram. Soc. 44 (1961) 420–421. https://doi.org/10.1111/j.1151-2916.1961.tb15476.x.

[4] R.A. Parker, Static Dielectric Constant of Rutile (TiO_2), 1.6-1060°K, Phys Rev. 124 (1961) 1719–1722. https://doi.org/10.1103/PhysRev.124.1719.

[5] W. Martienssen, H. Warlimont, eds., Springer Handbook of Condensed Matter and Materials Data, Springer Berlin, Heidelberg, 2005.

[6] P. de Rouffignac, A.P. Yousef, K.H. Kim, R.G. Gordon, ALD of Scandium Oxide from Scandium Tris(N , N ′ -diisopropylacetamidinate) and Water, Electrochem. Solid-State Lett. 9 (2006) F45. https://doi.org/10.1149/1.2191131.
